# Protective Effects of Allicin on Acute Myocardial Infarction in Rats *via* Hydrogen Sulfide-mediated Regulation of Coronary Arterial Vasomotor Function and Myocardial Calcium Transport

**DOI:** 10.3389/fphar.2021.752244

**Published:** 2022-01-03

**Authors:** Tianwei Cui, Weiyu Liu, Chenghao Yu, Jianxun Ren, Yikui Li, Xiaolu Shi, Qiuyan Li, Jinyan Zhang

**Affiliations:** ^1^ Institute of Basic Medical Sciences, Xiyuan Hospital, China Academy of Chinese Medical Sciences, Beijing, China; ^2^ Department of Reproductive Medicine, The First Affiliated Hospital of Henan University of Chinese Medicine, Zhengzhou, China; ^3^ Health Prevention Department, Xiyuan Hospital, China Academy of Chinese Medical Sciences, Beijing, China; ^4^ Beijing Key Laboratory of TCM Basic Research on Prevention and Treatment of Major Disease, Experimental Research Center, China Academy of Chinese Medical Sciences, Beijing, China; ^5^ Department of General Medicine, Xiyuan Hospital, China Academy of Chinese Medical Sciences, Beijing, China

**Keywords:** allicin, myocardial infarction, hydrogen sulfide, calcium homeostasis, coronary artery

## Abstract

Acute myocardial infarction (AMI) is a condition with high morbidity and mortality, for which effective treatments are lacking. Allicin has been reported to exert therapeutic effects on AMI, but the underlying mechanisms of its action have not been fully elucidated. To investigate this, a rat model of AMI was generated by ligating the left anterior descending branch of the coronary artery. DL-propargylglycine (PAG), a specific hydrogen sulfide (H_2_S) synthetase inhibitor, was used to examine the effects of allicin on H_2_S production. Isolated coronary arteries and cardiomyocytes were assessed for vascular reactivity and cellular Ca^2+^ transport using a multiwire myography system and a cell-contraction-ion detection system, respectively. Allicin administration improved cardiac function and myocardial pathology, reduced myocardial enzyme levels, and increased H_2_S and H_2_S synthetase levels. Allicin administration resulted in concentration-dependent effects on coronary artery dilation, which were mediated by receptor-dependent Ca^2+^ channels, ATP-sensitive K^+^ channels, and sarcoplasmic reticulum (SR) Ca^2+^ release induced by the ryanodine receptor. Allicin administration improved Ca^2+^ homeostasis in cardiomyocytes by increasing cardiomyocyte contraction, Ca^2+^ transient amplitude, myofilament sensitivity, and SR Ca^2+^ content. Allicin also enhanced Ca^2+^ uptake via SR Ca^2+^-ATPase and Ca^2+^ removal via the Na^+^/Ca^2+^ exchanger, and it reduced SR Ca^2+^ leakage. Notably, the protective effects of allicin were partially attenuated by blockade of H_2_S production with PAG. Our findings provide novel evidence that allicin-induced production of H_2_S mediates coronary artery dilation and regulation of Ca^2+^ homeostasis in AMI. Our study presents a novel mechanistic insight into the anti-AMI effects of allicin and highlights the therapeutic potential of this compound.

## 1 Introduction

Acute myocardial infarction (AMI) is a condition with high morbidity and mortality (McAloon et al., 2016). Despite the substantial technological and pharmacological developments of the recent years, the steady increase in the incidence of AMI and its poor prognosis remain significant clinical problems. Therefore, there is an urgent need to develop novel therapeutic strategies for preventing AMI. In this regard, traditional Chinese medicine (TCM) offers various advantages including the ability to target multiple biological pathways, low toxicity and costs, and fewer side effects (Liu et al., 2011).

Allicin, the main pharmacologically active ingredient in crushed raw garlic cloves (Lawson and Hunsaker, 2018), has various cardioprotective properties (Mocayar Marón et al., 2020), including blood pressure reduction, blood lipid regulation, atherosclerosis prevention, and myocardium protection against AMI. Nevertheless, the biological mechanisms underlying the protective action of allicin against AMI have not been fully elucidated. Studies have reported that in a rat model of AMI, allicin reduced oxidative stress injury and apoptosis by modulating the JNK signaling pathway in cardiomyocytes (Xu et al., 2020), and inhibited inflammation by relieving myocardial ischemia-reperfusion injury (Liu et al., 2019). We previously demonstrated that allicin exerted anti-fibrotic and anti-apoptotic effects in the myocardium, thereby ameliorating cardiac dysfunction in a rat model of AMI (Ma et al., 2017). Extensive evidence supports the importance of coronary artery tension, which maintains sufficient blood supply to the myocardium, in AMI injury (Uren et al., 1994). In addition, Ca^2+^ homeostasis, which is regulated by Ca^2+^-induced excitation-contraction coupling, is a critical determinant of cardiac contractile function. In fact, Ca^2+^ dyshomeostasis may lead to impaired systolic-diastolic function of cardiomyocytes after AMI (Zhang et al., 2021). Therefore, we speculated that the anti-AMI effects of allicin might be related to the coronary vasomotor function and Ca^2+^ transport in cardiomyocytes.

Hydrogen sulfide (H_2_S) plays a crucial role in cardiovascular homeostasis. In the human body, H_2_S production is predominantly catalyzed by cystathionine-γ-lyase (CSE), cystathionine-β-synthase (CBS), and 3-mercaptopyruvate sulfurtransferase (3-MST). To date, most scholars have proposed that CSE is the primary H_2_S-producing enzyme in the cardiovascular system (Singh and Banerjee, 2011; Leigh et al., 2016). H_2_S induces vasodilation, promotes angiogenesis, and regulates endothelial cell migration and inflammatory pathways (Li S. et al., 2017; Wang et al., 2021). Studies have showed that H_2_S can mediate the vasoactivity of garlic (Benavides et al., 2007), and that diallyl disulfide, a compound found in garlic, is a H_2_S-donor in both a cell-free system and vascular cells (Martelli et al., 2013; Martelli et al., 2020). As a sulfur compound, allicin has also been suggested to exert cardiovascular effects via the production of H_2_S *in vivo* (Wang et al., 2010). We previously demonstrated that allicin reduced blood pressure by promoting vasodilation in spontaneously hypertensive rats by inducing H_2_S production (Cui et al., 2020), a finding that was consistent with the literature. The present study aimed to evaluate the beneficial effects of allicin in a rat model of AMI and to elucidate the mechanisms related to H_2_S production. We hypothesized that allicin might protect against AMI injury in rats by inducing coronary artery vasodilation and regulating Ca^2+^ homeostasis by favoring H_2_S production.

## 2 Materials and Methods

### 2.1 Chemicals and Reagents

Allicin was provided by Xinjiang Ailexin Pharmaceutical Co., Ltd. (Batch No. 20190428, Xinjiang, China). DL-propargylglycine (PAG, inhibitor of the hydrogen sulfide synthetase, cystathionine-gamma-lyase [CSE]) was purchased from Shanghai Yuanye Bio-Technology (S18M7L11462, Shanghai, China). Diltiazem (a Ca^2+^-channel blocker) was purchased from Tianjin Tanabe Pharmaceutical Co., Ltd. (Batch No. 1905029, Tianjin, China). Potassium chloride (KCl), 5-hydroxytryptamine (5-HT), endothelin-1 (ET-1), tetraethylamine (TEA, inhibitor of Ca^2+^-sensitive potassium channels), 4-aminopyridine (4-AP, inhibitor of voltage-dependent potassium channels), barium chloride (BaCl_2_, inhibitor of inwardly rectifying potassium channels) and glibenclamide (Glib, inhibitor of ATP-sensitive potassium channels) were purchased from Sigma-Aldrich (St. Louis, MO, United States). All other reagents were of analytical purity.

### 2.2 Measurement of the Effects of Allicin on Acute Myocardial Infarction and Involvement of Hydrogen Sulfide

#### 2.2.1 Animals

Male 8-week-old Sprague Dawley (SD) rats (weighing 200–220 g) were utilized in the present study.

#### 2.2.2 Induction of Myocardial Infarction, Animal Grouping, and Treatment

The AMI model was generated via left anterior descending coronary artery (LAD) ligation in SD rats. Briefly, rats were anesthetized with 1% pentobarbital sodium. After left thoracotomy, the heart was exteriorized, and the LAD was ligated approximately 2 mm below the left atrium with a 6–0 silk suture. AMI was confirmed by elevation of the ST segment on an electrocardiogram and bulging of the relevant segment of the left ventricle (LV). In the sham group, the suture was removed without tying, and no infarction was generated. After establishment of the AMI model, rats were divided into six groups (*n* = 14–16 per group) by a random number table: sham, model, diltiazem (8.1 mg/kg), allicin (14 mg/kg), allicin (7 mg/kg), and allicin (14 mg/kg) + PAG (32 mg/kg) groups (Cui et al., 2020). All groups received intraperitoneal injection once a day for 7 days.

#### 2.2.3 Echocardiography and Myocardial Staining

Cardiac function was assessed using a Vevo 3100 echocardiography system (Visual sonics Inc, Toronto, Canada). Rats were anesthetized with 1.5–2% isoflurane via continuous inhalation and warmed on a heated pad (37°C). Ultrasound transmission gel was applied to the chest, and echocardiography (M-mode and B-mode imaging) was performed. The LV internal diameter and thickness of the anterior wall at end-diastole (LVID d, LVAW d) and end-systole (LVID s, LVAW s), as well as LV fractional shortening (FS), ejection fraction (EF), and stroke volume (SV) were measured in each rat in a blinded manner. All values were averaged using three to five cardiac cycles per rat. Rat hearts were harvested after 1% pentobarbital sodium overdose via intraperitoneal injection and sliced into five sections of 1-mm thickness across the left ventricular long axis under the ligature. To identify the infarction area, heart slices were incubated with nitro-blue tetrazolium chloride (Sigma-Aldrich) for 3 min at 22 ± 2°C. Infarction areas were measured using Image-Pro Plus software (Version 6.0; Media Cybernetics, Silver Springs, MD, United States) and presented as a percentage of infarct area to ventricular area or total area.

#### 2.2.4 Serum cTnI, LDH, and CSE Levels

Before the rats were sacrificed, blood samples were collected from the abdominal aorta. Serum was incubated at 22 ± 2°C for 30 min and centrifuged at 975.87 ×g for 10 min. The supernatant was collected for determination of serum cardiac troponin I (cTnI), lactate dehydrogenase (LDH), and CSE levels. Levels of serum cTnI, LDH, and CSE were separately quantified with commercially available cTnI (Medical Discovery Leader, Beijing, China, 159632), LDH (Medical Discovery Leader, Beijing, China, 164752), and CSE ELISA kits (Bluegene, Shanghai, China, E02C0834); antibody and chromogenic agent were added according to the manufacturer’s instructions. Absorbance was measured at 450 nm, detected by a microplate tester. Levels of LDH, cTnI, and CSE were calculated according to the standard curve (Chen et al., 2019; Li J. et al., 2020; Wu et al., 2020).

#### 2.2.5 Immunofluorescence Assay of Myocardial CSE

The border zone of myocardial infarction tissues were fixed with 4% (v/v) paraformaldehyde and incubated with dimethylbenzene for 30 min before serum blocking for 60 min. Specimens were incubated with CSE antibody (Proteintech, Wuhan, China, 12217-1-AP) for 24 h at 4°C prior to incubation with goat anti-rabbit IgG (H + L) fluorescein isothiocyanate-conjugated polyclonal antibody (20200321, Bai Aotong Experimental Materials Center, Luoyang, China) in the dark at 37°C for 60 min. After washing specimens in phosphate buffer solution, nuclei were stained with 4′, 6-diamidino-2-phenylindole (Sigma-Aldrich). Images were obtained using an upright fluorescence microscope (DM-LFS, Leica, MH, Germany) under ×400 magnification.

#### 2.2.6 Hydrogen Sulfide Levels

Levels of H_2_S in serum and the border zone of myocardial infarction tissue were measured using methylene blue spectrophotometry at 665 nm according to manufacturer’s instructions (Nanjing Jiancheng Bioengineering Institute, Nanjing, China).

#### 2.2.7 Histologic Examination

Hearts were harvested, weighed, washed in phosphate buffer solution, fixed in 4% paraformaldehyde overnight, and embedded in paraffin. Each paraffin-embedded heart was cut into 4-µm thick sections through the infarction area and stained with hematoxylin and eosin (H&E) for morphological observation. Specimens were stained with Masson’s trichrome stain to evaluate collagen volume. Sections were imaged using a stereomicroscope (Olympus SZ61, Tokyo, Japan).

### 2.3 Allicin Treatment of Isolated Coronary Arteries

#### 2.3.1 Preparation of Rat Coronary Arterial Rings

The coronary arterial rings (diameter: 100–300 µm) of SD rats were then isolated and placed in a cold Krebs buffer [composition (mM): NaCl, 119; KCl, 4.6; CaCl_2_, 1.5; NaH_2_PO_4_, 1.2; MgCl_2_, 1.2; NaHCO_3_, 15; Glucose, 5.6; pH 7.4]. RCARs were prepared and performed as previously described (Cui et al., 2020).

#### 2.3.2 Allicin-Induced Vasodilation of Rat Coronary Arterial Rings

RCARs were contracted with KCl (6 × 10^–2^ M) until a plateau of contraction was reached. The rings were then divided equally into two groups (8 rings each): PAG or control. RCARs were incubated with PAG (10^–2^ M) or the same volume of saline, respectively, for 5 min. Both groups were treated with allicin (10^−5^–10^–4.2^ M) and cumulative concentration-response curves were obtained.

#### 2.3.3 Effect of Allicin on Ca^2+^ Channel-Induced Contraction

KCl mediates the opening of voltage-dependent Ca^2+^ channels (VDCCs), whereas U46619, 5-HT, and ET-1 mediate the opening of receptor-dependent Ca^2+^ channels (RDCCs). In this study, RCARs were divided into three groups (8 rings each): allicin, allicin + PAG, and control groups. Rings were incubated with allicin (10^–4.8^ M), allicin (10^–4.8^ M) + PAG (10^–2^ M), or the equivalent volume of saline, respectively, for 5 min. Cumulative concentration-response curves for KCl (10^–1.54^–10^–1.42^ M), U46619 (10^−8^–10^–5^ M), 5-HT (10^−8^–10^–4^ M), and ET-1 (10^−9^–10^–6^ M) were obtained.

#### 2.3.4 Effects of Potassium Pathway Inhibitors on Allicin-Induced Vasodilation

To investigate the contribution of Ca^2+^-sensitive potassium channels (K_Ca_), voltage-dependent potassium channels (Kv), inwardly rectifying potassium channels (K_ir_), and ATP-sensitive potassium channels (K_ATP_) to allicin-induced vasodilation, the corresponding inhibitors, TEA (10^–3^ M), 4-AP (10^–3^ M), BaCl_2_ (10^–5^ M) and Glib (10^–5^ M) were applied. RCARs were divided into two groups (8 rings each): control and inhibitor groups. The rings were incubated with the four respective inhibitors for each channel or the same volume of saline for 5 min. Cumulative concentration-response curves for allicin (10^−5^–10^–4.2^ M) were obtained.

If the maximum vasodilatory effect in the inhibitor group was lower than that in the control group, subsequent experiments were conducted, as follows. The rings were divided into three groups (8 rings each): inhibitor, PAG, and PAG + inhibitor groups. Cumulative concentration-response curves for allicin (10^−5^–10^–4.2^ M) were obtained as described above.

#### 2.3.5 Effect of Allicin on Caffeine-Induced Contraction

The grouping and intervention for RCARs were performed as described above (See “Effect of Allicin on Ca^2+^ Channel-induced Contraction” section). Contraction-response curves for caffeine (3 × 10^–2^ M) were obtained.

### 2.4 Effects of Allicin on Ca^2+^ Transport in Cardiomyocytes

#### 2.4.1 Measurement of Sarcomere Shortening and Cytosolic Ca^2+^ Transients

Isolated cardiomyocytes were loaded with 2 μM Fura-2 AM (Sigma-Aldrich) in the dark for 30 min at 22 ± 2°C. Cells were washed, resuspended twice in Tyrode’s solution (concentration in mM: 137.0 NaCl, 1.2 NaH_2_PO_4_, 5.0 KCl, 1.2 MgCl_2_, 10.0 HEPES, 10.0 glucose, and 1.2 CaCl_2_ [pH 7.4]), and placed in a cell chamber. Myocytes were stimulated to contract at a pacing frequency of 1 Hz with 4 ms of electrical stimulation. Myocytes were exposed to 340 or 380 nm excitation wavelengths, and the emitted fluorescent signal was detected at 510 nm. Sarcomere length and fluorescence intensity (a proxy of Ca^2+^ concentration) were synchronously recorded with a cell contraction-ion detection system (IonOptix, Westwood, MA, United States). Contractility parameters including amplitude, peak time, systolic half-time of decay (T_50_), diastolic T_50_, and myofilament sensitivity were measured. Ca^2+^ transient parameters including amplitude, maximum ascending and descending velocity, and Ca^2+^ decline time constant were also recorded.

#### 2.4.2 Measurement of Sarcoplasmic Reticulum Ca^2+^ Content

SR Ca^2+^ content is associated with caffeine-sensitive Ca^2+^ release (Santulli et al., 2017). Short puffs of 10 mM caffeine were applied to completely empty the SR, following a train of 1-Hz field stimulation to achieve steady-state SR Ca^2+^ loading in ventricular myocytes. SR Ca^2+^ content was assessed by measuring the amplitude of caffeine-elicited Ca^2+^ transients (△F/F_0_).

#### 2.4.3 Assessment of Ca^2+^ Removal

Rapid and continuous application of 10 mM caffeine was employed to induce SR Ca^2+^ release and assess the contribution of the Na^+^/Ca^2+^ exchanger (NCX) and slow transport systems (mitochondrial Ca^2+^ uniporter and sarcolemmal Ca^2+^-ATPase). The contribution of the slow transport system to Ca^2+^ removal is only 1% and is often overlooked (Puglisi et al., 2014). With continuous caffeine superfusion, a decrease in fluorescence (F340/380) indicates Ca^2+^ removal, which is predominantly attributable to the NCX. Ca^2+^ removal was predominantly achieved by sarco/endoplasmic reticulum Ca^2+^-ATPase (SERCA) uptake and NCX Ca^2+^ efflux with superfusion of Tyrode’s solution. Based on these factors, the time of SERCA-mediated Ca^2+^ removal was calculated (Tau).

#### 2.4.4 Ca^2+^ Leakage Assessment

Ca^2+^ leakage levels were assessed by perfusing myocytes with 1 mM tetracaine and reperfusing in 10 mM Na-, Ca-free Tyrode’s solution (containing Li^+^ and EGTA instead of Na^+^ and Ca^2+^). Levels of SR Ca^2+^ leakage were calculated based on the difference in cytosolic Ca^2+^ concentration before and after tetracaine perfusion.

### 2.5 Statistical Analysis

The SPSS statistical software (SPSS 20.0, IBM, Chicago, IL, United States) was used for statistical analysis. Vasodilation and vasocontraction are expressed as the percentage of precontraction amplitude. The negative logarithms of the concentration that produced the half-maximal effect (pEC_50_) and maximum relaxation (R_max_) or contraction (E_max_) were determined using concentration negative logarithm-effect curves. The differences in vasomotor responses to allicin, as well as in the levels of KCl, 5-HT, U46619, and ET-1 were compared by a two-way analysis of variance (ANOVA), with a *post hoc* Bonferroni test for group comparison. Statistical significance was determined through a one-way ANOVA with Dunnett’s test for multiple-group comparisons in other experiments. ^*^ *p* < 0.05, ^**^ *p* < 0.01 vs. sham group, ^△^ *p* < 0.05, ^△△^ *p* < 0.01 vs. AMI model group, ^#^ *p* < 0.05, ^##^ *p* < 0.01 vs. allicin 14 mg/kg group in all figures. All data are presented as the mean ± S.E.M.

## 3 Results

### 3.1 Effects of Allicin on Acute Myocardial Infarction and the Involvement of Hydrogen Sulfide

#### 3.1.1 Cardiac Function

**FIGURE 1:**
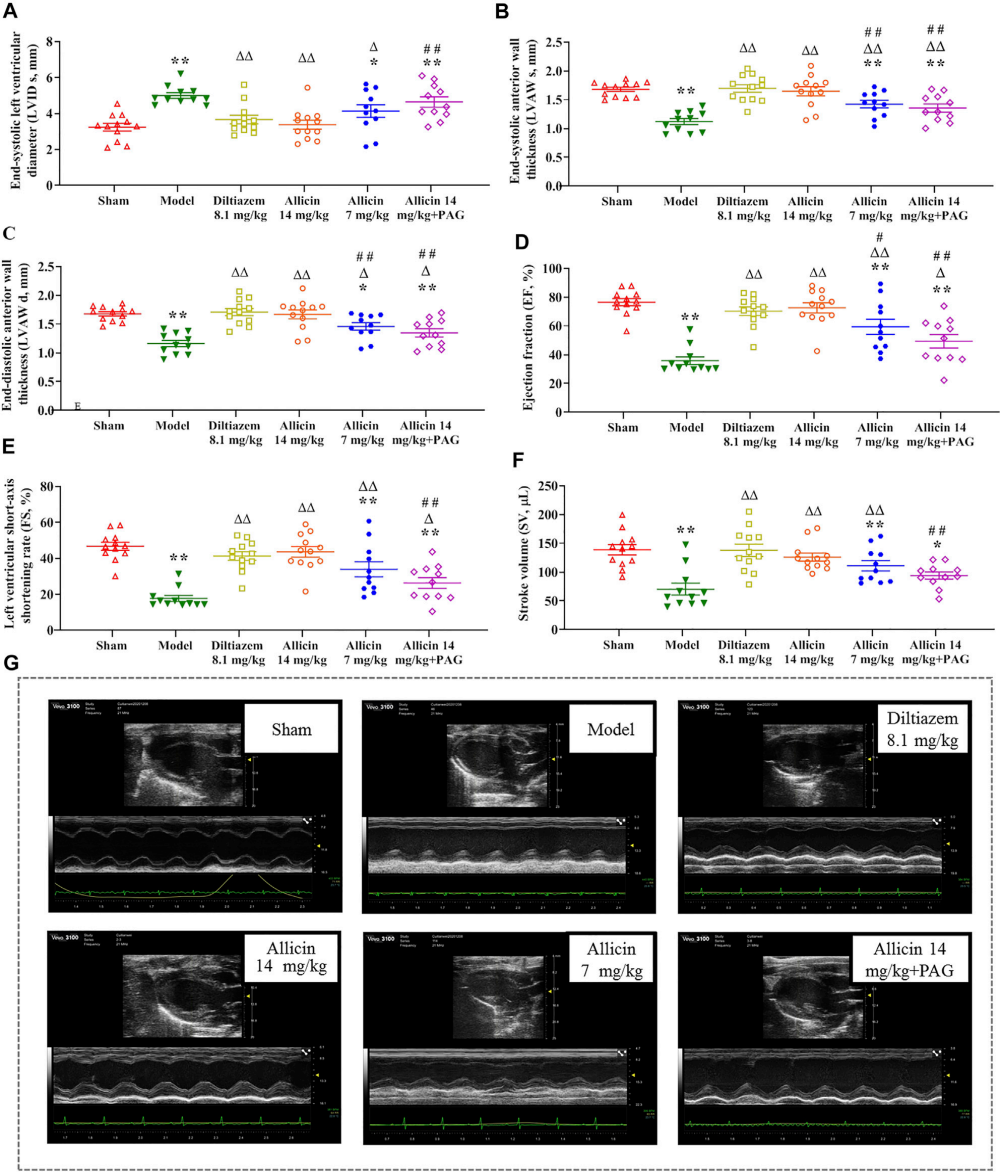
Allicin improved cardiac function in AMI model rats. **(A‒F)** The statistical scatter plots of **(A)** LVID s, **(B)** LVAW s, **(C)** LVAW d, **(D)** EF, **(E)** FS, and **(F)** SV in the experimental and control groups. **(G)** Representative images of ultrasonic function in experimental and control groups. Data are expressed as mean ± S.E.M (*n* = 11–12). ^*^ *p* < 0.05, ^**^ *p* < 0.01 vs. control; ^△^ *p* < 0.05, ^△△^ *p* < 0.01 vs. the Model group; ^#^ *p* < 0.05, ^##^ *p* < 0.01 vs. the Allicin 14 mg/kg group. Abbreviations: LVID s, left ventricular internal diameter end systole; LVAW s, left ventricular end-systolic anterior wall thickness; LVAW d, left ventricular end-diastolic anterior wall thickness; EF, ejection fraction; FS, functional shortening; SV, stroke volume; PAG, DL-propargylglycine.

Ventricular size and function were measured to assess the effect of allicin treatment. There was no statistical difference in LVID d among the groups. LVID s was significantly greater in the model group than in the sham group (Figure 1A), whereas the values of LVAW s, LVAW d, EF, FS, and SV were significantly lower in the model group than in the sham group. On the contrary, LVID s was significantly lower, and LVAW s, LVAW d, EF, FS, and SV were significantly higher, in the diltiazem 8.1 mg/kg and allicin 14 mg/kg groups than in the model group. LVID s was significantly higher, and LVAW s, LVAW d, EF, FS, and SV were significantly lower in the allicin 14 mg/kg + PAG group than in the allicin 14 mg/kg group (Figures 1B‒F). Hence, as shown in Figure 1G, we found that allicin significantly improved the cardiac function of AMI rats, and PAG partially weakened this effect.

#### 3.1.2 Myocardial Infarction Size

**FIGURE 2:**
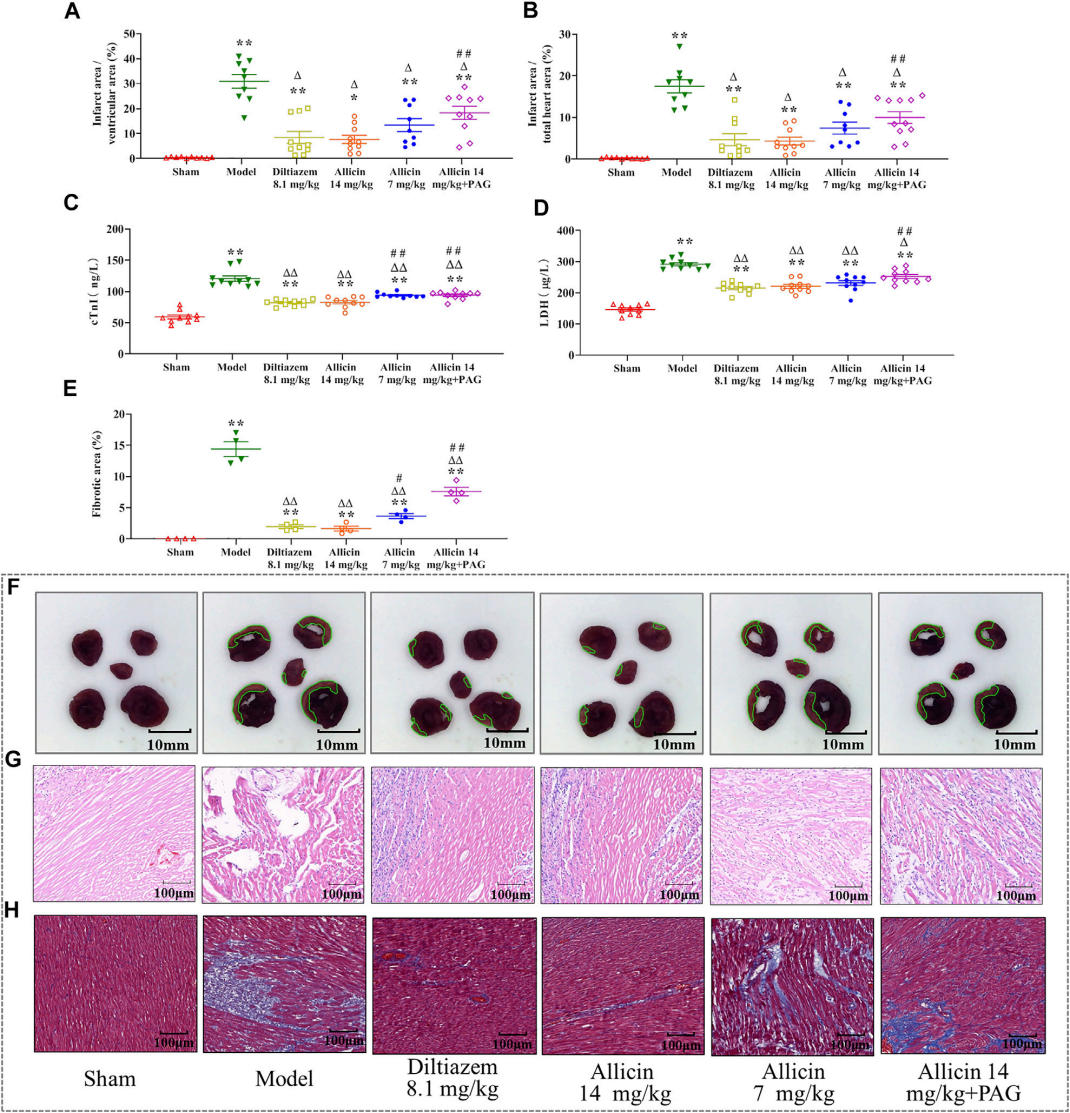
Allicin reduced myocardial infarction area and improved morphological changes in myocardial tissue. **(A,B)** Percentage of myocardial infarction area over the ventricular and total heart area, respectively (*n* = 9–11). **(C,D)** Effects of allicin on serum cTnI and LDH levels (*n* = 9–11). **(E)** Percentage of myocardial fibrosis area stained with Masson’s trichrome staining (*n* = 4). **(F)** Representative myocardial infarction areas in each group (*n* = 9–11). **(G,H)** Effects of allicin on the pathological morphology of the myocardium in rats with acute myocardial infarction by H&E staining and Masson staining (*n* = 9–11), magnification ×200. Data are expressed as mean ± S.E.M. ^*^ *p* < 0.05, ^**^ *p* < 0.01 vs. control; ^△^ *p* < 0.05, ^△△^ *p* < 0.01 vs. the Model group; ^#^ *p* < 0.05, ^##^ *p* < 0.01 vs. the Allicin 14 mg/kg group. Abbreviations: LDH, lactate dehydrogenase; cTnI, cardiac troponin; PAG, DL-propargylglycine.

The percentages of myocardial infarction area to ventricular area (ITV) and total heart area (ITT) were significantly lower in the sham group (0.42 ± 0.26% and 0.23 ± 0.16%, respectively) than in the model group (30.89 ± 8.26% and 17.49 ± 4.75%, respectively). ITV and ITT were significantly reduced by treatment with 14 mg/kg of allicin (7.67 ± 5.15% and 4.33 ± 2.84%, respectively) and 8.1 mg/kg of diltiazem (8.27 ± 7.65% and 4.67 ± 4.57%, respectively). However, in the allicin 14 mg/kg + PAG group, ITV and ITT were significantly higher (17.57 ± 8.17% and 9.97 ± 4.63%, respectively) than in the allicin 14 mg/kg group, but significantly lower than in the model group (Figures 2A,B). Hence, allicin significantly decreased the myocardial infarction area of AMI rats, and PAG partially weakened the effect of allicin (Figure 2E).

#### 3.1.3 Serum cTnI and LDH Levels

cTnI and LDH levels were significantly higher in the model group than in the sham group (cTnI: 120.53 ± 13.93 ng/L vs. 59.39 ± 9.81 ng/L; LDH: 291.81 ± 15.91 µg/L vs. 146.62 ± 16.20 µg/L), indicating that the model successfully mimicked AMI conditions. Levels of cTnI and LDH were significantly lower in the diltiazem 8.1 mg/kg group (82.12 ± 5.09 ng/L and 215.06 ± 16.16 µg/L, respectively), allicin 14 mg/kg group (82.82 ± 8.30 ng/L and 220.77 ± 19.85 µg/L, respectively), allicin 7 mg/kg group (94.04 ± 3.84 ng/L and 231.55 ± 24.56 µg/L, respectively), and allicin 14 mg/kg + PAG group (94.14 ± 6.28 ng/L and 252.20 ± 20.53 µg/L, respectively) than in the model group. cTnI and LDH levels were significantly higher in the allicin 14 mg/kg + PAG group than in the allicin 14 mg/kg group. These data suggest that treatment with diltiazem or allicin significantly alleviated the changes in cardiac function induced by AMI, and PAG partially reversed this effect (Figures 2C,D).

#### 3.1.4 Pathological Morphology

H&E and Masson staining revealed that in the sham group, the structure of cardiomyocytes remained intact, the transverse and fiber striations of the myocardium were clear, and cells were arranged regularly. No degeneration, necrosis, hemorrhage, inflammatory cell infiltration, or collagen deposition was observed in the sham group. In the model group, the arrangement of myocardial fibers was disordered, swollen, and disjointed; the septum of the fiber bundles was widened; transverse lines of cells were absent, striations were disordered, normal cell structure was disrupted; and extensive cardiomyocyte necrosis, interstitial vascular hyperplasia, hyperemia, edema, inflammatory cell infiltration, and collagen deposition were observed. These features are typical of myocardial infarction. In the allicin 14 mg/kg and diltiazem 8.1 mg/kg groups, the arrangement of cardiomyocytes was slightly disordered, myocardial fibers were neatly arranged, the interstitium was broadened, the septum of the fiber bundles was not significantly widened, no cell necrosis was observed, and minimal cardiomyocyte edema and collagen deposition were noted. Hence, the degree of pathological damage observed in the allicin 7 mg/kg and allicin 14 mg/kg + PAG treatment groups was intermediate compared with that noted in the model and allicin 14 mg/kg groups (Figures 2F,G).

#### 3.1.5 Hydrogen Sulfide Levels in Serum and Myocardial Tissue

**FIGURE 3:**
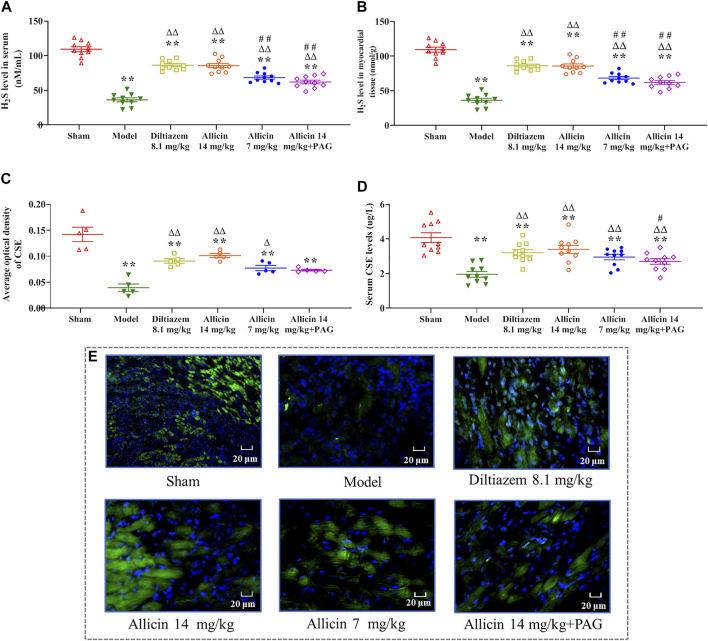
Allicin increased H_2_S and CSE levels. **(A)** Serum H_2_S levels; **(B)** H_2_S levels in myocardial tissue; **(C)** CSE levels in myocardial tissue; **(D)** Serum CSE levels. **(E)** Representative micrographs of heart sections from AMI rats stained to visualize the CSE enzyme (green) and nuclei (DAPI; blue), magnification ×400. Data are expressed as mean ± S.E.M (*n* = 5–12). ^*^ *p* < 0.05, ^**^ *p* < 0.01 vs. control; ^△^ *p* < 0.05, ^△△^ *p* < 0.01 vs. the Model group; ^#^ *p* < 0.05, ^##^ *p* < 0.01 vs. the Allicin 14 mg/kg group. Abbreviations: CSE, cystathionine-γ-lyase; AMI, acute myocardial infarction; PAG, DL-propargylglycine.

The levels of the H_2_S in serum and the border zone of the myocardial infarction tissues were significantly lower in the model group (36.23 ± 8.96 nM/ml and 342.18 ± 48.77 nM/g, respectively) than in the sham group (109.39 ± 11.44 nM/ml and 682.93 ± 56.83 nM/g, respectively) (Figures 3A,B). On the other hand, the levels of H_2_S in the serum and myocardial tissue were significantly higher in the diltiazem 8.1 mg/kg (86.21 ± 7.03 nM/ml and 543.91 ± 49.15 nM/g, respectively), allicin 14 mg/kg (85.73 ± 9.06 nM/ml and 563.91 ± 34.28 nM/g, respectively), allicin 7 mg/kg (68.35 ± 7.18 nM/ml and 469.82 ± 48.25 nM/g, respectively), and allicin 14 mg/kg + PAG groups (62.00 ± 8.48 nM/ml and 490.53 ± 47.75 nM/g, respectively) than in the model group, suggesting that these treatments alleviated myocardial infarction symptoms. High levels of allicin were more effective than lower levels, as H_2_S serum and tissue levels were significantly lower in the allicin 7 mg/kg and allicin 14 mg/kg + PAG groups than in the allicin 14 mg/kg group. Finally, the levels of H_2_S in the serum and myocardial tissue were significantly higher in the allicin 14 mg/kg group than in the model group.

#### 3.1.6 CSE Levels

Immunofluorescence analysis revealed that the average optical density (AOD) of CSE in myocardial tissue was significantly lower in the model group (0.039 ± 0.014) than in the sham group (0.141 ± 0.028). CSE showed a significantly higher level in the diltiazem 8.1 mg/kg (0.091 ± 0.009), allicin 14 mg/kg (0.101 ± 0.008), allicin 7 mg/kg (0.077 ± 0.010), and allicin 14 mg/kg + PAG groups (0.073 ± 0.003) than in the model group. On the other hand, CSE levels tended to be lower in the allicin 7 mg/kg and allicin 14 mg/kg + PAG groups than in the allicin 14 mg/kg group, but this trend did not reach statistical significance (Figure 3C). Similarly, serum CSE levels were significantly lower in the model group than in the sham group (CSE: 1.95 ± 0.52 µg/L vs. 4.07 ± 0.88 µg/L). Serum CSE levels were significantly higher in all treatment groups than in the model group. CSE levels were significantly lower in the allicin 14 mg/kg + PAG group than in the allicin 14 mg/kg group (Figure 3D). Figure 3E suggests that in rats with AMI, allicin treatment improved CSE levels partly via inducing H_2_S production.

### 3.2 Allicin-Regulated Coronary Artery Vasomotor Function

#### 3.2.1 Vasodilatory Effects of Allicin on Rat Coronary Arteries *via* Hydrogen Sulfide

**FIGURE 4:**
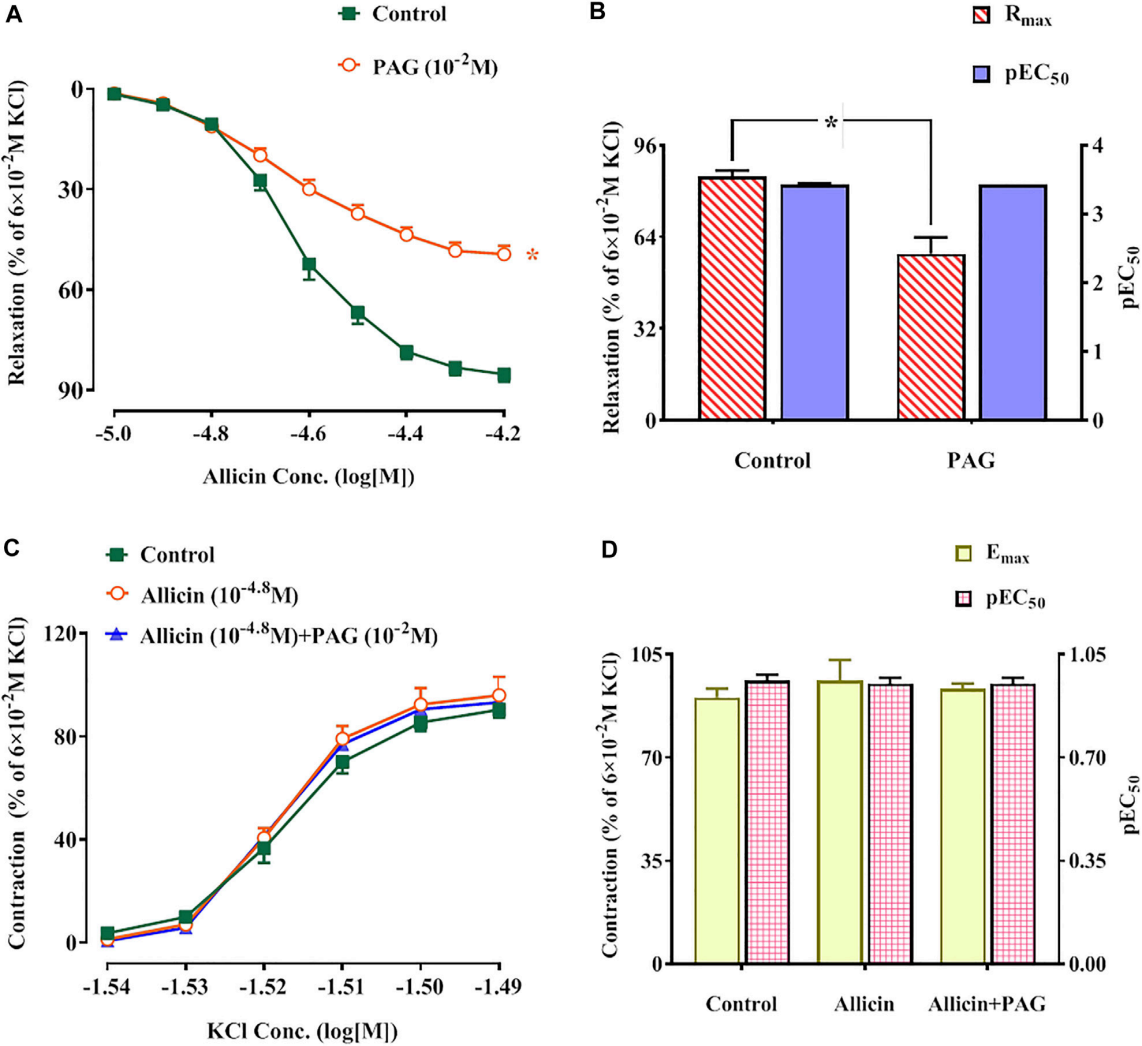
**(A,B)** Allicin induced coronary artery dilation in rats, while PAG inhibited this effect. **(A)** Diastolic effect curves of allicin adding saline and PAG, respectively; **(B)** R_max_ and pEC_50_ of diastolic effect curve of allicin. **(C,D)** Effects of allicin on dose-response curves of KCl-induced contraction in rat coronary arteries and the intervention effects of PAG. **(C)** Diastolic effect curve of KCl; **(D)** R_max_ and pEC_50_ of diastolic effect curve of KCl. Data are presented as mean ± S.E.M (8 rings from 4‒8 rats for each group). ^*^ *p* < 0.05 vs. control. Abbreviations: PAG, DL-propargylglycine; pEC_50_, half-maximal effect; R_max,_ maximum relaxation; E_max_, maximum contraction.

Allicin-induced dilation in rat coronary arteries precontracted with KCl in a dose-dependent manner. The maximum vasodilation reached 85.11 ± 2.11% of the pre-contraction amplitude. After the application of PAG, the maximum relaxation induced by allicin was 49.37 ± 6.94%, which was significantly lower than that in the control group (Figures 4A,B). The inhibitory rate of PAG on the vasodilation effect of allicin was 42.4%. There was no significant difference in pEC_50_ between the two groups.

#### 3.2.2 Allicin-Mediated Inhibition of Dose-dependent Potassium Chloride-Induced Contraction in Rat Coronary Arteries

KCl induced contractions in rat coronary arteries in a dose-dependent manner. No significant changes in E_max_ and pEC_50_ of KCl-induced concentration-contraction curves were observed after administration of allicin or allicin + PAG (Figures 4C,D).

#### 3.2.3 Effects of Allicin on Receptor-Dependent Ca^2+^ Channel Agonist Dose-Response Curves in Rat Coronary Arteries

**FIGURE 5:**
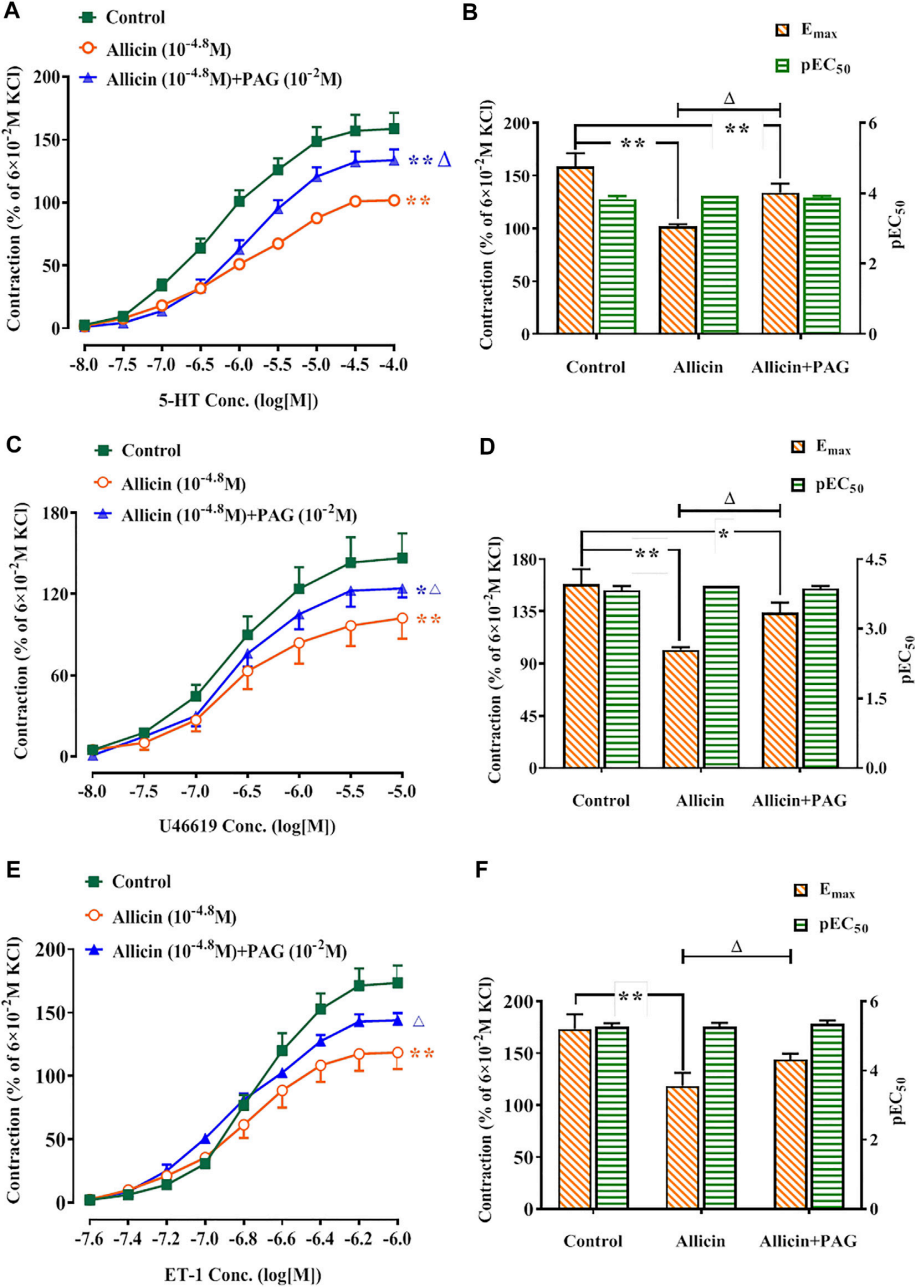
Allicin inhibited 5-HT, U46619, and ET-1-induced contractions in rat coronary arteries and the intervention effects of PAG. **(A,C,E)** Diastolic effect curves of 5-HT, U46619, and ET-1; **(B,D,F)** R_max_ and pEC_50_ of diastolic effect curves of 5-HT, U46619, and ET-1. Data are presented as mean ± SEM (8 rings in each group from 4‒8 rats). ^*^ *p* < 0.05, ^**^ *p* < 0.01 vs. control; ^△^ *p* < 0.05 vs. the Allicin group. Abbreviations: PAG, DL-propargylglycine; pEC_50_, half-maximal effect; R_max,_ maximum relaxation; E_max_, maximum contraction; 5-HT, 5-hydroxytryptamine; ET-1, endothelin 1.

Following administration of allicin, the E_max_ values of the concentration-contraction curves induced by 5-HT, U46619, and ET-1 were 101.86 ± 2.16%, 102.19 ± 15.24%, and 120.77 ± 13.98%, respectively. These values were significantly lower than those of the control group (158.73 ± 12.63%, 146.56 ± 18.23%, and 173.51 ± 14.09%, respectively). Following administration of allicin + PAG, the E_max_ values of the concentration-contraction curves induced by 5-HT, U46619, and ET-1 were 133.91 ± 8.65%, 124.09 ± 6.64%, and 144.02 ± 5.22%. These values were significantly lower than those of the control group but were significantly higher than those of the allicin group (Figure 5).

#### 3.2.4 The Weakening Effect of a K^+^ Pathway Inhibitor on Allicin-Induced Vasodilation of Rat Coronary Arteries

**FIGURE 6:**
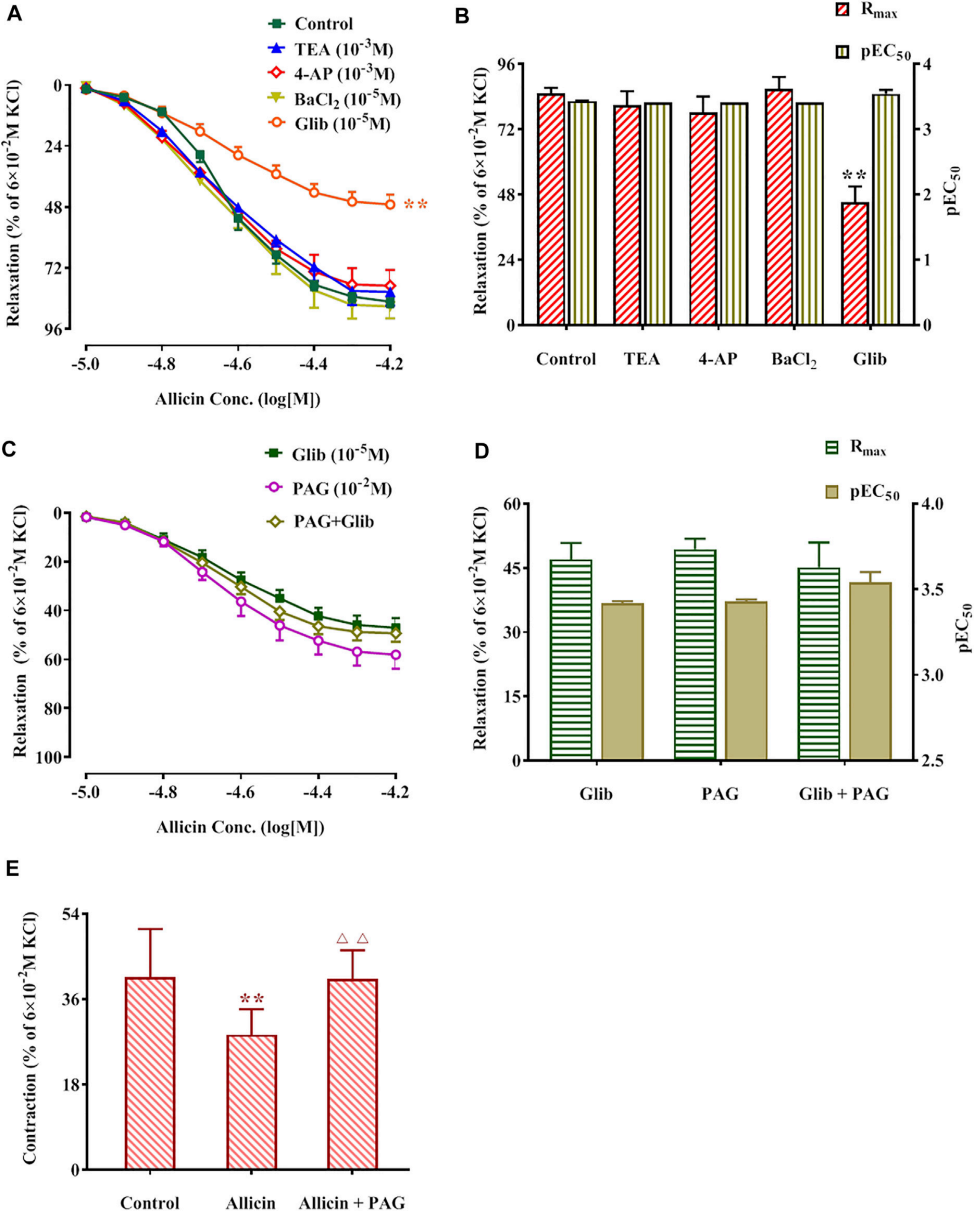
Potassium pathway inhibitors reduced allicin-induced diastolic effects in rat coronary arteries via H_2_S and allicin inhibited caffeine-induced coronary artery contraction. **(A,B)** The diastolic effect curves of allicin and R_max_ and pEC_50_ after adding saline and four potassium pathway inhibitors respectively; **(C,D)** The diastolic effect curves of allicin and R_max_ and pEC_50_ after adding PAG or/and Glib, respectively; **(E)** Effect of allicin on caffeine-induced contraction. Data are presented as mean ± S.E.M (8 rings in each group from 4‒8 rats). ^**^ *p* < 0.01 vs. control; ^△△^ *p* < 0.01 vs. the Allicin group. Abbreviations: PAG, DL-propargylglycine; Glib, glibenclamide; 4-AP, 4-aminopyridine; TEA, tetraethylamine; pEC_50_, half-maximal effect; R_max,_ maximum relaxation.

The R_max_ of allicin in the TEA, 4-AP, and BaCl_2_ groups was not significantly different compared with that of the control group (82.90 ± 6.22%), whereas the R_max_ of allicin was significantly lower in the Glib group (46.98 ± 3.86%) than in the control group. No significant differences were observed in the R_max_ of allicin among the PAG, PAG + Glib, and Glib groups. Altogether, these data suggest that the response to allicin is mediated by receptor-dependent, rather than voltage-dependent, Ca^2+^ channels (Figures 6A‒D).

#### 3.2.5 The Inhibitory Effect of Allicin on Caffeine-Induced Coronary Artery Contraction in Ca^2+^-free Solution

Caffeine (3 × 10^–2^ M) induced a transient and rapid contraction of rat coronary arteries in Ca^2+^-free solution. The contraction amplitude was 40.70 ± 10.09% of the contraction induced by KCl (6 × 10^–2^ M). After administration of allicin, the contraction amplitude induced by caffeine was 28.45 ± 5.42%, which was significantly lower than that in the control group. Caffeine-induced contraction amplitude was significantly higher in the allicin + PAG group (40.28 ± 6.05%) than in the allicin group (Figure 6E).

### 3.3 Allicin-Mediated Enhancement of Cardiomyocyte Sarcomere Shortening and Ca^2+^ Transients at the Infarct Border

#### 3.3.1 Cardiomyocyte Sarcomere Shortening

**FIGURE 7:**
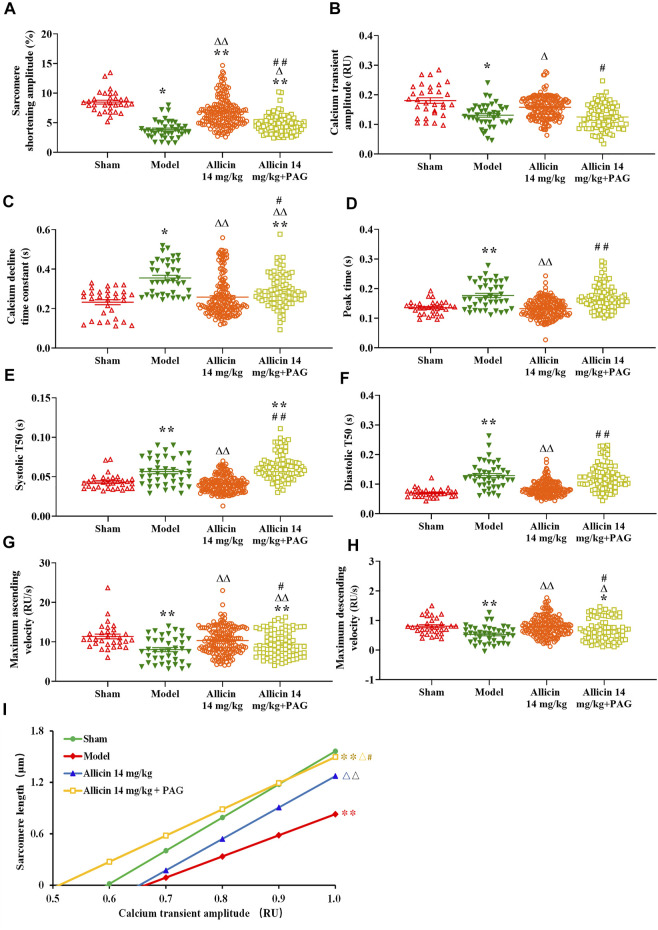
Allicin enhanced cardiomyocyte sarcomere shortening and Ca^2+^ transients. **(A)** Contraction amplitude; **(B)** Calcium transient amplitude; **(C)** Ca^2+^ decline time constant; **(D)** Peak time; **(E)** Systolic T_50_; **(F)** Diastolic T_50_; **(G)** Maximum ascending and descending velocities; **(H)** Maximum decline time constant; **(I)** Myofilament sensitivity. Data are presented as mean ± S.E.M (*n* = 31–175 cardiomyocytes from 3‒5 rats). ^*^ *p* < 0.05, ^**^ *p* < 0.01 vs. control; ^△^ *p* < 0.05, ^△△^ *p* < 0.01 vs. the Model group; ^#^ *p* < 0.05, ^##^ *p* < 0.01 vs. the Allicin 14 mg/kg group. Abbreviations: PAG, DL-propargylglycine; T_50_, half-time of decay.

Figure 7 shows that the contraction amplitude (3.85 ± 1.49%) was significantly lower, whereas peak time (0.17 ± 0.04 s), systolic T_50_ (0.056 ± 0.017 s), and diastolic T_50_ (0.128 ± 0.044 s) were significantly longer in the model group than in the sham group (8.47 ± 1.80%, 0.13 ± 0.02 s, 0.043 ± 0.009 s, and 0.069 ± 0.014 s, respectively). Contraction amplitude in the allicin 14 mg/kg (6.76 ± 2.43%) and allicin 14 mg/kg + PAG groups (4.65 ± 1.53%) was higher, whereas peak time, systolic T_50_, and diastolic T_50_ in the allicin 14 mg/kg group (0.13 ± 0.02 s, 0.041 ± 0.010 s, 0.081 ± 0.033 s) was significantly lower than that in the model group. The addition of PAG significantly reduced the contraction amplitude in the allicin 14 mg/kg + PAG group (4.65 ± 1.53%) compared with the allicin 14 mg/kg group. Similarly, peak time, systolic T_50_, and diastolic T_50_ were significantly longer in the allicin 14 mg/kg + PAG group (0.16 ± 0.04 s, 0.062 ± 0.016 s, and 0.122 ± 0.046 s, respectively) than in the allicin 14 mg/kg group, indicating that PAG significantly alleviated the effects of allicin.

#### 3.3.2 Ca^2+^ Transients

AMI significantly reduced the amplitude, maximum ascending velocity, and maximum descending velocity of Ca^2+^ transients in the model group (0.13 ± 0.03 RU [ratio unit], 8.02 ± 3.20 RU/s, and 0.52 ± 0.28 RU/s, respectively) compared with the sham group (0.18 ± 0.05 RU, 11.36 ± 3.27 RU/s, and 0.80 ± 0.28 RU/s, respectively). The Ca^2+^ decline time constant in the model group (0.35 ± 0.08 s) was significantly longer than that in the sham group (0.23 ± 0.07 s) as well. Ca^2+^ transient amplitude in the allicin 14 mg/kg group (0.15 ± 0.03 RU) and maximum ascending and descending velocities of Ca^2+^ transients in the allicin 14 mg/kg (10.26 ± 3.42 RU/s and 0.79 ± 0.34 RU/s, respectively) and allicin 14 mg/kg + PAG groups (9.39 ± 3.26 RU/s and 0.68 ± 0.38 RU/s, respectively) were significantly higher than in the model group. The Ca^2+^ decline time constants in the allicin 14 mg/kg (0.25 ± 0.10 s) and allicin 14 mg/kg + PAG groups (0.28 ± 0.08 s) were significantly shorter than in the model group. Compared with the allicin 14 mg/kg group, the allicin 14 mg/kg + PAG group exhibited significantly lower Ca^2+^ transient amplitude and maximum ascending and descending velocities of Ca^2+^ transients, as well as significantly longer Ca^2+^ decline time constant (Figure 7).

#### 3.3.3 Myofilament Sensitivity

Diastolic curves with good linear relationships were obtained from the nonlinear fitting curve between sarcomere length and Ca^2+^ transient amplitude. The slope of this curve was calculated to reflect myofilament sensitivity. Myofilament sensitivity of cardiomyocytes in the model group (2.46 ± 0.77) was significantly lower than in the sham group (3.86 ± 1.76). By contrast, myofilament sensitivity in the allicin 14 mg/kg and allicin 14 mg/kg + PAG groups was significantly higher than that in the model group. Myofilament sensitivity was significantly lower in the allicin 14 mg/kg + PAG group than in the allicin 14 mg/kg group (Figure 7I).

#### 3.3.4 Sarco/Endoplasmic Reticulum Ca^2+^-ATPase-Mediated Ca^2+^ Reabsorption and Na^+^/Ca^2+^ Exchanger-Mediated Ca^2+^ Efflux

**FIGURE 8:**
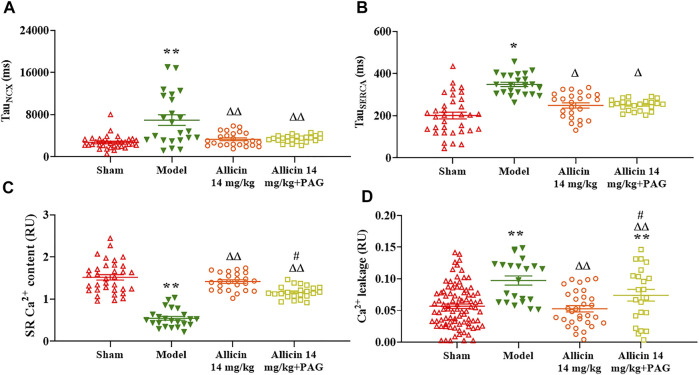
Effect of allicin on myocardial Ca^2+^ transport. **(A,B)** Allicin enhanced SERCA reabsorption and NCX-induced calcium efflux in cardiomyocytes. **(C,D)** Allicin increased SR Ca^2+^ content and reduced Ca^2+^ leakage in cardiomyocytes. *n* = 22–89 cardiomyocytes from 3‒5 rats. Data are presented as mean ± S.E.M. ^*^ *p* < 0.05, ^**^ *p* < 0.01 vs. control; ^△^ *p* < 0.05, ^△△^ *p* < 0.01 vs. the Model group; ^#^ *p* < 0.05 vs. the Allicin 14 mg/kg group. Abbreviations: PAG, DL-propargylglycine; SERCA, sarco/endoplasmic reticulum Ca^2+^-ATPase; NCX, Na^+^/Ca^2+^ exchanger; SR, sarcoplasmic reticulum.

Tau_NCX_ and Tau_SERCA_ were significantly higher in the model group (6986.2 ± 5238.6 ms and 349.5 ± 63.5 ms, respectively) than in the sham group (2732.3 ± 1275.1 ms and 200.7 ± 93.6 ms, respectively). Compared with the model group, the Tau_NCX_ and Tau_SERCA_ of the allicin 14 mg/kg group (3202.9 ± 1473.1 ms and 246.4 ± 63.1 ms, respectively) and allicin 14 mg/kg + PAG group (3584.4 ± 768.0 ms and 246.4 ± 29.1 ms, respectively) were both significantly decreased. No significant differences were observed in Tau_NCX_ and Tau_SERCA_ between the allicin 14 mg/kg and allicin 14 mg/kg + PAG groups (Figures 8A,B).

#### 3.3.5 Sarcoplasmic Reticulum Ca^2+^ Content and Ca^2+^ Leakage

The SR Ca^2+^ content of myocardial cells was threefold lower in the model group (0.53 ± 0.16 RU) than in the sham group (1.51 ± 0.37 RU). On the other hand, the allicin 14 mg/kg (1.43 ± 0.18 RU) and allicin 14 mg/kg + PAG (1.17 ± 0.13 RU) treatments induced significantly higher SR Ca^2+^ content when compared with the model group. SR Ca^2+^ content was significantly lower in the allicin 14 mg/kg + PAG group than in the allicin 14 mg/kg group.

Ca^2+^ leakage was significantly higher in the model group (0.097 ± 0.033 RU) than in the sham group (0.052 ± 0.040 RU), whereas allicin 14 mg/kg (0.056 ± 0.05 RU) and allicin 14 mg/kg + PAG (0.073 ± 0.044 RU) administration significantly decreased leakage compared with the model group. Ca^2+^ leakage was significantly higher in the allicin 14 mg/kg + PAG group than in the allicin 14 mg/kg group (Figures 8C,D).

## 4 Discussion

AMI is a prevalent cardiovascular event (Lu et al., 2015). Despite early revascularization, timely medical therapy, and up-to-date mechanical circulatory support, AMI prognosis remains poor and patient mortality remains high. TCM, which is underscored by a rich 2000–3000-year history of medical theories concerning disease etiology and treatments, has been in the public eye due its therapeutic promise for AMI, among other conditions. Here, we investigated the therapeutic effects of allicin, a component of garlic, in a rat model of AMI. We demonstrated that allicin exerted anti-AMI effects, including improvements in cardiac function as well as reduction in infarct size and serum cTnI and LDH levels. In parallel, microscopic observations revealed that allicin significantly improved the detrimental morphological changes observed in myocardial tissue after AMI, supporting the protective effects of allicin against cardiac lesions and myocardial cell necrosis.

Garlic, as a food source and medicinal plant, is widely known for its cardiovascular properties (Zhou et al., 2021). Allicin is well-established as the main pharmacological component of garlic. Allicin is an enzymatic product of alliin and alliinase, and is produced when raw garlic cloves undergo cell rupture. Extensive evidence supports the cardioprotective effects of allicin. For instance, allicin protected rats against AMI and myocardial ischemia reperfusion injury by suppressing inflammation, fibrosis, apoptosis, and oxidative stress (Liu et al., 2019). We previously demonstrated that allicin exerted anti-AMI effects (Ma et al., 2017). This study builds upon our previous findings and provides insight into the mechanisms underlying the anti-AMI effects of allicin.

Patients who survive AMI frequently develop systolic heart failure due to the infarct-induced loss of a functional myocardium and the remodeling of the LV. This process involves cardiomyocyte necrosis and hypertrophy, LV wall thinning, infarct expansion, and collagen accumulation (Wollert and Drexler, 2010). Further, serum levels of the cytosolic enzymes cTnI and LDH are significantly increased when myocardial ischemia and hypoxia occur (Goyal et al., 2010; Cordwell et al., 2012). In the event of AMI, myocardial circulatory perfusion becomes insufficient, leading to ischemia and hypoxia of the myocardial tissue, structural and functional damage to cardiomyocytes, irreversible cellular necrosis, and serious damage to cardiac contractile function. Therefore, increasing the blood supply to coronary arteries and improving the function of cardiomyocytes are key to effectively prevent and treat adverse clinical events after AMI.

In AMI, coronary microvascular dysfunction results in the insufficient supply of blood and oxygen and may affect myocardial function at rest and during stress (Michelsen et al., 2018). Given the protective effects of allicin on AMI, elucidating the regulatory effects of this compound on the vasomotor function of coronary arteries in the context of AMI-induced damage would provide valuable mechanistic insight. In this study, we observed that allicin exerted concentration-dependent vasodilatory effects on rat coronary arteries. Given that Ca^2+^ concentration is a critical factor for vascular tone (Shen et al., 2009), we focused on Ca^2+^-related vascular tension. Intracellular Ca^2+^ concentration is predominantly increased by Ca^2+^ influx and/or Ca^2+^ release from the SR (Guan et al., 2019). Ca^2+^ influx involves the opening of VDCCs and RDCCs. Ligands such as 5-HT, thromboxane A_2_ (TXA_2_), and ET-1, by binding to their corresponding G protein-coupled receptors, are the main activators of RDCCs, whereas VDCCs are predominantly activated by cations, including K^+^. We demonstrated that allicin significantly inhibited the contractions induced by 5-HT, U46619, and ET-1 in rat coronary arteries, but it did not alter contractions induced by KCl, suggesting that RDCCs, rather than VDCCs, mediated the extracellular Ca^2+^ influx that contributed to vasodilation. Receptors mediating SR Ca^2+^ release include the inositol triphosphate and ryanodine receptors (RyRs), of which, the latter mediates more than 90% of SR Ca^2+^ release. In order to study the effects of allicin on RyR-mediated calcium release, we used caffeine in Ca^2+^-free solution, which increased the intracellular Ca^2+^ concentration by completely activating the RyRs in the SR (Garcia et al., 2019). Our findings indicated that allicin significantly inhibited caffeine-induced coronary artery contractions, suggesting that allicin-induced vasodilation of coronary arteries was associated with inhibition of the SR Ca^2+^ release mediated by RyRs.

The opening of K^+^ channels, which leads to cell membrane hyperpolarization by promoting intracellular K^+^ outflow, is a way to decrease intracellular Ca^2+^ concentration and induce vasodilation. To date, four types of K^+^ channels with different activation mechanisms have been identified: these are the K_V_, K_Ca_, K_ir_, and K_ATP_ channels (Lorigo et al., 2020). These channels inhibit the activation of VDCCs on the cell membrane, thus reducing extracellular Ca^2+^ influx. Further, they increase intracellular Ca^2+^ efflux by stimulating the NCX, thus reducing intracellular Ca^2+^ and causing vasodilation. To investigate the involvement of K^+^ channels in allicin-induced vasodilation, we applied 4-AP, TEA, BaCl_2_, and Glib separately on coronary arteries to observe their effects on allicin-induced vasodilation. Only Glib application significantly inhibited allicin-induced vasodilation, suggesting that the vasodilatory effects of allicin on coronary arteries were at least partly attributed to K_ATP_ channel opening but did not involve other K^+^ channels.

Cardiomyocyte dysfunction is a direct consequence of AMI and leads to a decrease in cardiomyocyte vitality, which eventually results in heart failure and even death. Ca^2+^-mediated excitation-contraction coupling is a requirement for correct cardiomyocyte contraction and relaxation (Palomeque et al., 2009; Lahiri et al., 2021). During cardiac systole, a small amount of extracellular Ca^2+^ enters the cytoplasm through L-type Ca^2+^ channels, which triggers the activation of the RyRs in the SR and leads to extensive Ca^2+^ release from the SR. Intracellular Ca^2+^ binds to troponin to cause cell contraction (Zhang et al., 2018). Intracellular Ca^2+^ can be rapidly recaptured by SERCA on the SR and transported out of cells through the NCX on the cell membrane, which causes cardiomyocytes to enter a diastolic state. Ca^2+^ transients reflect the rapid dynamic changes in cytoplasmic Ca^2+^ during cardiomyocyte contraction and relaxation, and the changes in velocity and amplitude represent changes in myocardial contractility. In addition, the sensitivity of cardiomyocyte myofilaments to Ca^2+^ affects the contractile function of cardiomyocytes. Under pathological conditions, there is a decrease in the amplitude and velocity of Ca^2+^ transients of cardiomyocytes, SR Ca^2+^ content, and myofilament sensitivity to Ca^2+^. This leads to a decrease in cardiomyocyte contraction and relaxation function (D.M. Bers, 2002; Piacentino et al., 2003). In this study, we observed that allicin significantly increased the contractile amplitude of cardiomyocytes, maximum release and reabsorption rate of Ca^2+^ transients, and myofilament sensitivity of cardiomyocytes. Allicin also decreased the peak time, systolic T_50_, diastolic T_50_, and elimination constant of Ca^2+^ transient time. Collectively, these findings indicated that allicin significantly improved the contraction and relaxation function of myocardial cells.

In pathological states, intracellular Ca^2+^ is not reabsorbed by SERCA or expelled by the NCX in a timely manner at the end of cardiomyocyte contraction. Rather, intracellular Ca^2+^ accumulates in the cytoplasm, which hinders the separation of thin and thick myofilaments and leads to myocardial diastolic dysfunction. On the other hand, under physiological conditions, the elimination of Ca^2+^ transients is predominantly mediated by SERCA recapture, Ca^2+^ efflux by the NCX, and slow transport systems during end-diastole. In this regard, the contribution of slow transport systems is less than 1% (Matthew et al., 2004). In order to investigate the SR Ca^2+^ content and Ca^2+^ efflux induced by the NCX, we continuously perfused coronary arteries with caffeine in a Ca^2+^-free environment, which completely opened the RyRs. This enabled all Ca^2+^ in the SR to be completely released and temporarily offset SERCA-induced Ca^2+^ reabsorption. Therefore, cytoplasmic Ca^2+^ concentration reflected the SR Ca^2+^ content, and the elimination of Ca^2+^ transients was considered to be predominantly accomplished by the NCX. We demonstrated that allicin significantly enhanced SERCA recapture and increased Ca^2+^ extrusion by the NCX, and increased the SR Ca^2+^ content. Furthermore, RyR dysfunction is known to cause Ca^2+^ leakage during diastole (Fischer et al., 2013), which leads to intracellular Ca^2+^ overload, SR Ca^2+^ content decrease, and eventually, abnormal cell contraction (Sheibani et al., 2017). Our data demonstrated that allicin significantly decreased Ca^2+^ leakage, suggesting its ability to regulate Ca^2+^ homeostasis.

H_2_S functions as a gasotransmitter, similar to nitric oxide and carbon monoxide. H_2_S exerts various cardiovascular effects, including vasodilation, blood pressure reduction, and myocardium protection. Endogenous H_2_S, a potent vasodilator, is synthesized via the metabolic breakdown of L-cysteine by CSE, CBS, and 3-MST (Kanagy et al., 2017; Sheibani et al., 2017). Found predominantly in the cardiovascular system (Singh and Banerjee, 2011), CSE contributes to about 90% of total H_2_S production in organs that express all three enzymes (Leigh et al., 2016). In addition, it has been reported that CSE also exists as a circulating enzyme that is secreted by endothelial cells into the circulatory system, where it circulates as a member of the plasma proteome (Bearden et al., 2010). Allicin, as a sulfur-containing compound, may exert its cardiovascular effects by increasing the production of H_2_S. Furthermore, our previous research demonstrated that allicin reduced blood pressure by promoting H_2_S production. Here, we expand upon our previous findings by demonstrating that the anti-AMI effects of allicin are at least partially underpinned by the production of H_2_S mediated by CSE. In this regard, PAG, a CSE inhibitor, significantly attenuated, but did not completely abrogate, the positive effects that allicin exerts on AMI injury, coronary artery vasodilation, and calcium transport regulation in cardiomyocytes. In the present study, allicin increased the levels of H_2_S and CSE with or without PAG, but PAG reversed this effect, indicating that PAG partially impedes the allicin-induced production of H_2_S and CSE. Therefore, we speculate that allicin directly induces H_2_S production both directly and indirectly by increasing the levels of CSE. Several studies have shown that the mechanisms underlying H_2_S-induced vasorelaxation include the opening of K_ATP_ channels, closing of VDCC, and decreased concentration of intracellular Ca^2+^ (Holwerda et al., 2015; Hedegaard et al., 2016). It has been established that NaHS, an H_2_S-donor, improved diabetic cardiomyopathy by regulating the Ca^2+^-handling system in the SR (Cheng et al., 2016). Consistent with the literature, our results showed that the effects of allicin on Ca^2+^ and K^+^ currents are strictly superimposable to those exhibited by H_2_S. These findings suggest that H_2_S is involved in the anti-AMI effects of allicin, but other mechanisms are involved. Unexpectedly, our results showed that PAG weakened the effect of allicin on CSE production, causing a decrease in CSE levels. However, we are puzzled as to why PAG, an inhibitor of CSE activity, also reduces CSE level. After preliminary literature research, we found that quite a few studies reported that PAG could decrease CSE level (Li X. et al., 2017; Wang et al., 2019; Li Y. et al., 2020). The impact of PAG on CSE level, and the reason for such effect would require further in-depth studies.

An additional point worth noting is that most scholars believe that the CSE enzyme is mainly expressed in the cardiovascular system (Xu et al., 2014; Kolluru et al., 2015). Therefore, we selected a CSE enzyme inhibitor as a tool to explore the relationship between allicin-induced coronary vasodilation and H_2_S. The results showed that the CSE inhibitor significantly weakened the vasodilatory effects of allicin on isolated rat coronary arteries, indicating that CSE is indeed involved in allicin-induced vasodilation by mediating H_2_S production. However, a previous study on swine coronary arteries suggested that CBS was the most important enzyme for the production of H_2_S under hypoxic conditions, and that the vasodilatory contribution from CSE and 3-MST becomes apparent only upon inhibition of CBS (Donovan et al., 2017). Another study pointed out that 3-MST, rather than CSE, was the main enzyme expressed in the coronary arteries of rats and mice (Kuo et al., 2016). Therefore, the role of the three H_2_S-producing enzymes in coronary arteries is still debatable. In this study, we only evaluated the role of CSE, but not that of CBS or 3-MST; hence, this study cannot fully elucidate the role of each enzyme in the rat coronary artery. Our results showed that the CSE inhibitor inhibited allicin-induced vasodilation in coronary arteries by 42.4%, indicating that a considerable amount of CSE exists in rat coronary arteries, and that its role in inducing H_2_S production cannot be neglected, as previously suggested in some studies (Chai et al., 2015; Luo et al., 2021). Regarding the controversy over the dominant role of each H_2_S-producing enzyme in coronary arteries, four reasons could explain such discrepancies. First, the expression and activity of CSE, CBS, and 3-MST may vary across different species, and under different physiological or pathological conditions. Second, the levels of enzyme expression may not be proportional to the levels of activity, and indeed an inverse relationship may be present where lower expression results in higher activity. Third, enzyme activity may vary under different experimental conditions and methods, and lastly, the predominance of one H_2_S-producing enzyme over the others may differ with or without drug intervention. Therefore, further in-depth studies are needed to better understand the dominant role of CSE, CBS, and 3-MST in coronary arteries.

## 5 Conclusion

**FIGURE 9:**
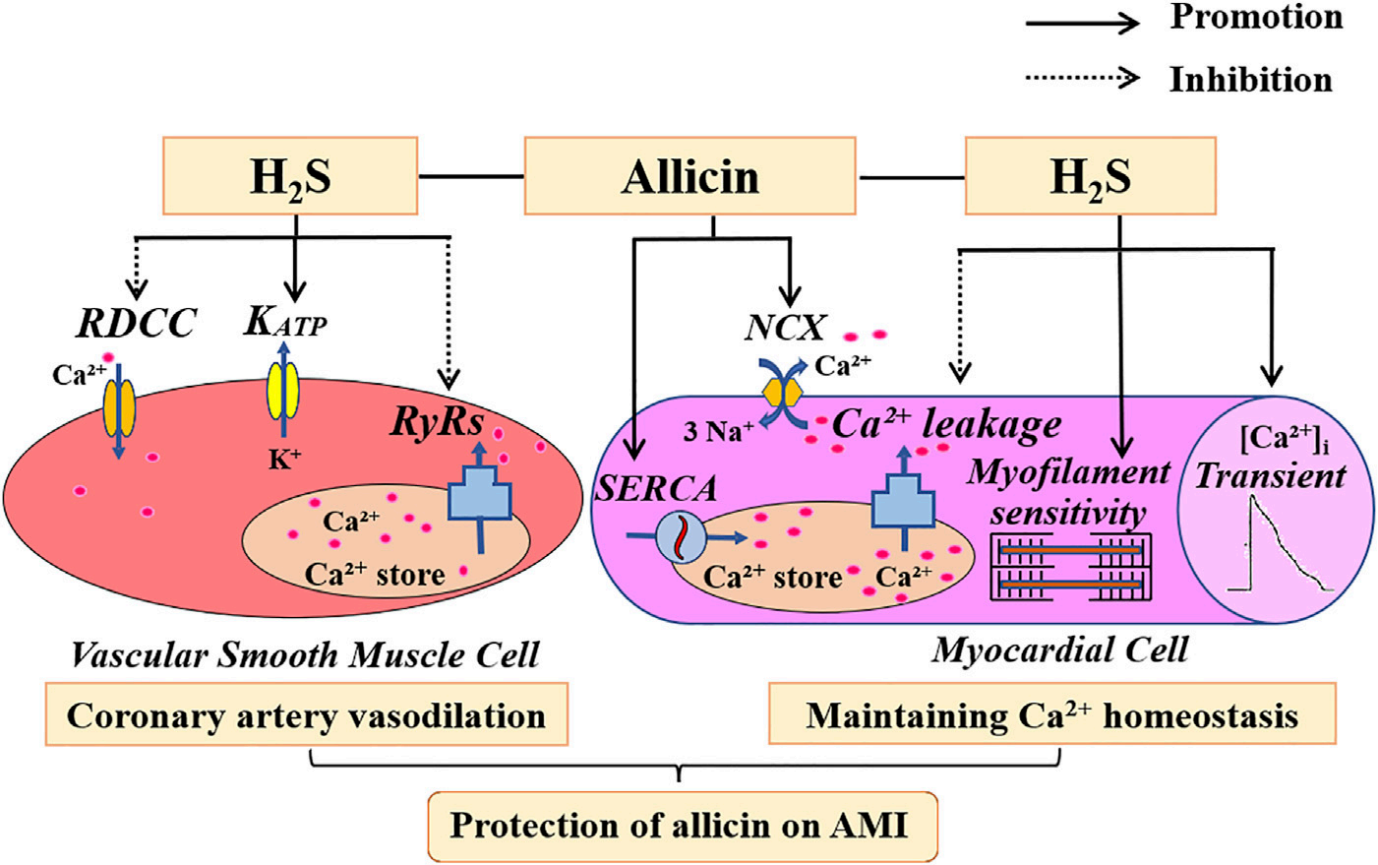
The potential mechanism of action of allicin in an AMI rat model. Allicin administration resulted in concentration-dependent effects on coronary artery dilation, which were mediated by RDCC, K_ATP_, and RyRs. Allicin administration improved Ca^2+^ homeostasis in the cardiomyocytes of rats with AMI by increasing cardiomyocyte contraction, Ca^2+^ transient amplitude, myofilament sensitivity, and SR Ca^2+^ content. Allicin also enhanced the Ca^2+^ uptake induced by SERCA and the Ca^2+^ removal induced by the NCX, and reduced Ca^2+^ leakage. H_2_S is partially involved in the anti-AMI effects of allicin. Abbreviations: RDCC, receptor-dependent Ca^2+^ channels; K_ATP_, ATP-sensitive potassium channels; RyRs, ryanodine receptors; SERCA, sarco/endoplasmic reticulum Ca^2+^-ATPase; NCX, Na^+^/Ca^2+^ exchanger; AMI, acute myocardial infarction.

Our study demonstrates that allicin may exert cardioprotective effects in a rat model of AMI injury by inducing coronary artery vasodilation and maintaining Ca^2+^ homeostasis via H_2_S production. Allicin induced the vasodilation of coronary arteries via favoring H_2_S production by inhibiting the opening of RDCC, promoting the opening of K_ATP_, and decreasing the Ca^2+^ release induced by the RyRs. Allicin regulated Ca^2+^ homeostasis by increasing cardiomyocyte contraction, Ca^2+^ transient amplitude, myofilament sensitivity, and SR Ca^2+^ content, and reducing SR Ca^2+^ leakage via H_2_S production. Moreover, allicin enhanced the Ca^2+^ uptake induced by SERCA and Ca^2+^ removal induced by the NCX, in which H_2_S is not involved. (Figure 9) This new understanding of the mechanisms underpinning the therapeutic effects of allicin will facilitate the development of effective therapeutic modalities for cardiac rehabilitation in humans.

## Data Availability

The raw data supporting the conclusions of this article will be made available by the authors, without undue reservation.
